# Development of model web-server for crop variety identification using throughput SNP genotyping data

**DOI:** 10.1038/s41598-019-41204-2

**Published:** 2019-03-26

**Authors:** Rajender Singh, M. A. Iquebal, C. N. Mishra, Sarika Jaiswal, Deepender Kumar, Nishu Raghav, Surinder Paul, Sonia Sheoran, Pradeep Sharma, Arun Gupta, Vinod Tiwari, U. B. Angadi, Neeraj Kumar, Anil Rai, G. P. Singh, Dinesh Kumar, Ratan Tiwari

**Affiliations:** 1 ICAR-Indian Institute of Wheat & Barley Research, Karnal, 132001 Haryana India; 2 ICAR- Indian Agricultural Statistics Research Institute, New Delhi, 110012 India

## Abstract

Crop varieties or genotypes of a given species are pivotal for agricultural production and ownership, management and improvement of their germplasm is a great challenge. Its morphological identification requires time, cost and descriptors are often compromised statistically due to phenotypic plasticity. Development of DNA based signature of varieties can overcome these limitations. There is a global need to implement world trade organization (WTO) and intellectual property rights (IPR) guidelines of Plant Breeders Rights (PBR) where DUS (distinctness, uniformity and stability) testing can be supplemented by DNA profile. Universalization and minimization of SNP number without compromising identification accuracy is the major challenge in development of varietal profile by rapid genotype assay. Besides this, there is no server-based approach reducing computational skill with global accessibility of referral phenotypic and genotypic data. We report world’s first model web server for crop variety identification using >350 Indian wheat varieties and Axiom 35 K SNP chip data. Standard filtering and linkage disequilibrium approach were used to develop varietal signature in Linux using HTML, Java, PHP and MySQL with provision of QR code generator to facilitate bar-coding. Phylogenetic tree constructed by selected SNPs confirms six major trait based clusters of varieties and their pedigree. Our user friendly server based tool, VIS*Ta* (Variety Identification System of *Triticum aestivum*) (http://webtom.cabgrid.res.in/vista) can be used in DUS testing having dispute resolution of sovereignty and access benefit sharing (ABS) issues. This model approach can be used in other crops with pan-global level management of crop germplasm in endeavour of crop productivity.

## Introduction

Out of 250–300 thousands edible plant species, only 5% are relevant to agriculture. Among these, three species, namely maize, rice and wheat fulfills 60% of the caloric needs of humans^1^. Crop variety resources are pivotal for agricultural production, their selection and management is an integral part of variety development^2^. The identification of large number of plant varieties solely on the basis of morphological characters is difficult due to growth stage specificity and phenotypic plasticity^3^. Development of DNA based signature profiles of released varieties to compare with candidate variety or future variety is required.

In sweep of globalization having WTO and IPR guidelines, it is imperative to have rapid and highly reliable method of plant variety identification system (VIS). Traditional approaches of VIS were exclusively dependent on morphological characters (descriptors) having compromised precision and time consuming. For example, in potato varieties having 50 characters, 12 of them are time dependent with growth stage, which can never be rapid. Such limitations are encountered in almost every crop^3^. To protect intellectual property (IP) of variety by granting statutory Plant Breeders Rights (PBR), it requires testing of distinctness, uniformity and stability (DUS) called DUS testing. It has limitations like growth stage specificity, environmental influence, phenotypic plasticity, ineffectiveness over large collections, lack of rapidity besides statistical compromise in the values of descriptors. If VIS tool is available to breeders, growers and the general industry, it would be instrumental in rapid identification, germplasm registration and traceability of the produce of concerned crop variety. Such tool can also prevent economic loss of variety developer’s investment, which may happen due to deceptive indication of variety status by unauthorized user of variety. The majority of the crop diversity is present in developing countries and global use of germplasm is often encountered with sovereignty and access benefit sharing (ABS) issues^4^.

This is a great challenge for taxonomy, law, ethical consideration and technology to establish varietal status. For example, wheat varietal disputes over varieties Nap-Hal and Galahad having quality trait for biscuits, flour and dough making are globally best known example^5^ where Patent EP 445929 was revoked. Molecular markers especially protein profiling of glutenin and gliadins to supplement DUS features are reported more than a decade ago, where centralized publically accessible data on referral varieties can be used as “last resort” to resolve the dispute^6^.

Use of next generation sequence based approaches has emerged as a powerful tool for characterization of varieties based on genomic sequence differences. Single Nucleotide Polymorphisms (SNPs) are the most common type of genomic variations which represent differences in a single DNA building block. Therefore, SNP markers relate to their ease of data management along with their flexibility, speed and cost effectiveness. In fact >50 SNP arrays are available for >25 various crop genomes^7^. There is no dearth of molecular data for variety identification but VIS still has the challenge of universalization of SNPs across variety, SNP minimization, development of genotype assay, multiplexing, computational skill, online accessibility of molecular data along with variety descriptor. Pan-global approach of development of VIS is still lacking.

Till now there is no web-based methodology/ approach for varietal identification of any crop using throughput SNP data. There is a need to develop a user friendly server based tool where no computational skill is required and user can obtain variety identification results with its DUS features. Such tool can further supplement DUS varietal testing, which would be more relevant in era of globalization where transboundary movement of germplasm often leads to sovereignty disputes. We report world’s first model web server of any crop for variety identification using Indian wheat varieties and its Axiom 35 K SNP data as an example.

## Materials and Methods

### DUS Phenotyping of model crop wheat

Extensive phenotyping was done to generate data of wheat DUS features to confirm the varietal status of a panel of 368 Indian spring wheat genotypes to be used in the study. The panel constituted were represented by 116 released varieties, 45 registered genetic stocks, 117 advanced breeding lines and 90 Indian landraces. Among 36 DUS features 28 were qualitative or categorical and 8 were quantitative. The DUS features represents 8 plant description traits, 9 ear characters, 6 flag leaf attributes, 6 glume features, 4 grain appearance and 3 grain quality traits. The characters were recorded according to DUS test guidelines framed by Protection of Plant Varieties and Farmers’ Rights Authority, India (http://plantauthority.gov.in/pdf/GBread%20Wheat.pdf). The quantitative characteristic, except days to heading and test weight, were recorded from ten plants in each of three replications. The days to heading was recorded on plot basis while thousand grains were randomly selected and weighed for test weight. The qualitative characters were recorded by the visual assessment on individual plant or on parts of the plants of individual genotypes. Out of 27 traits of latest 2017 UPOV guidelines 24 traits are covered in our recording barring three, namely, (i) straw: pith in cross section, (ii) Apical rachis segment: area of haireness on convex surface and (iii) lower glume: area of hairiness on internal surface.

### DNA extraction and SNP genotyping

DNA was isolated from the leaves of two week old seedlings using CTAB method^8,9^. The DNA samples were genotyped using Axiom^®^ Wheat Breeder’s Genotyping Array (Affymetrix UK Ltd, UK).

### SNP data analysis

Filtering of SNPs was done with parameters: call rate (<95%), monomorphic, >10% missing value, MAF (<0.05) and heterozygosity (>1%). Remaining SNPs were used for further analysis. Statistical values, namely, minor allele frequency (MAF), gene diversity, heterozygosity and Polymorphic Information Content (PIC) for each SNP were estimated using PowerMarker v3.25^10^. In development of any crop variety identification methodology/system, the smallest number of markers discriminating all or maximum number of varieties in the panel are most desirable. Such approach of SNP minimization reduces the cost of genotyping without compromising the identification accuracy^11,12^. In fact 2–3 SNP/per chromosome are enough for variety identification^13^. In order to minimize number of SNPs, pairwise locus Linkage Disequilibrium (LD) was estimated by TASSEL 3.0^14^. Further SNPs were selected based on following criteria: (1) SNP markers with PIC value more than 0.35, (2) the SNP marker were selected based on the consensus map derived from five mapping populations (Allen *et al*., 2016) where each SNP was mapped in at least in two mapping populations (http://www.cerealsdb.uk.net/cerealgenomics/CerealsDB/axiom_download.php), (3) atleast two markers from each chromosome were selected for distinguishing genotypes and to differentiate closely related genotypes, more SNP markers were included (4) SNP markers were selected in such a way that they are not closely linked to each other except two markers each on chromosomes 2A, 6A and 7B. Graphical representation of the distribution of SNP markers on 21 chromosomes was done using GGT software 2.0^15^. The position of SNP markers in terms of genetic distance (cM) were based on consensus genetic map generated from five mapping populations^14^. With the objective of not compromising the utility value of the SNP panel in resolving differences between close genotypes, markers differentiating those genotypes were also included. The genetic distances across the genotypes and neighbor-joining (NJ) tree based on Nei 1983 were calculated using PowerMarker v3.25 and NJ trees visualized using MEGA 4^16^. BA codes were retrieved from CerealsDB (http://www.cerealsdb.uk.net/cerealgenomics/CerealsDB/indexNEW.php) which corresponds to Affymetrix Axiom array SNP markers (http://www.cerealsdb.uk.net/cerealgenomics/CerealsDB/axiom_download.php). Further these BA codes were used to search EnsemblPlants *T. aestivum* database (http://plants.ensembl.org/) to find the position of variants.

### Putative candidate gene analysis

To find the putative candidate genes for the reported SNPs, we performed a BLASTn search of NCBI database (http://www.ncbi.nlm.nih.gov/) with the SNP sequences. The putative candidate genes identified from BLASTn were further searched in UniProtKB (https://www.uniprot.org/) to find the putative biological functions which were supported by existing literature. In case of hypothetical proteins, they were characterized by predicting its genes and translated proteins using protein BLAST (PAM algorithm having lenient stringency).

### Generation of 2-D barcode

For accessions being used in this study, 2D barcode was generated using online tool (available at www.barcode-generator.org). Each accession and its genotype based SNP barcode was used as input to generate corresponding 2D barcode. Once the barcode was generated, it was scanned for the confirmation of information used for creating the 2D barcode.

### Development of web-based variety identification system for wheat

Variety Identification System for *Triticum aestivum* (VIS*Ta*) was developed in a LINUX operating system using HTML and JAVA as client-side scripting, PHP as server side scripting language and MYSQL as the RDBMS to store genotype data of 368 wheat verities with 54 SNPs markers. This is launched on Apache internet server. VIS*Ta* tool has provision to enter query data, search and measure the distance of query data against database along with the presentation of results in tabular and graphical mode. This web-based tool is easy to use and allows access to varietal identification and validation along with database through a user-friendly web- browser. The database is designed and developed on relational database concept. VIS*Ta* is intended to store and manage genotype data of 368 varieties of wheat and fast data retrieval required through web-interfaces. HTML and java scripting languages have been used for client side operations such as manual data entry, loading data from a text file and QR code image file. The sample data is kept in the server for users for ease of understanding and its implementation. PHP server-side language has been used for database connectivity, retrieval of data and calculation of distance measure of query data against database. HTML and Java scripting language has been used for illustration of results in tabular and graphical form. For most convenient remote location use, a mobile app has also been developed. We opted for QR code for information matrix due to its advantages, namely, being two-dimensional, it can hold more information (both horizontally and vertically), and thus minimizing space needed for printing in leveling of wheat seeds/produce. This will have more convenience and ease in labeling of germplasm especially while managing in wet-lab and crop-field both. Such approach has advantage of error-free, machine based retrieval of information and effective transmission required in germplasm management. It can be read in 360 degrees thus more convenient. It can also accommodate symbol/logo etc. of variety holder/ organization, if required.

## Results and Discussion

### Minimization of SNPs for crop variety DNA signature development

Of 35143 SNPs obtained with Axiom® Wheat Breeder’s Array, 6041 SNPs were removed having a call rate <95% leaving 29102 SNPs for downstream analysis. Out of these, 3.54% (1031) SNPs were monomorphic and 1.54% (448) showed >10% missing values. These SNPs were removed from the dataset. In addition, 7680 (26.39%) SNPs with a MAF of <0.05 and 3878 (13.33%) SNPs with >1% heterozygosity were also excluded from the dataset. Remaining 16065 (55.2%) SNPs were included for further analysis. The genotypes included in the study were released varieties, registered genetic stocks, advanced breeding lines and landraces. The MAF of SNPs ranged from 0.05 to 0.5 with average 0.2619. The gene diversity across 16065 loci ranged from 0.095 to 0.5 with average 0.3537. The mean PIC value of SNPs was 0.2837 with a range of 0.0905 to 0.375.

**Figure 1:**
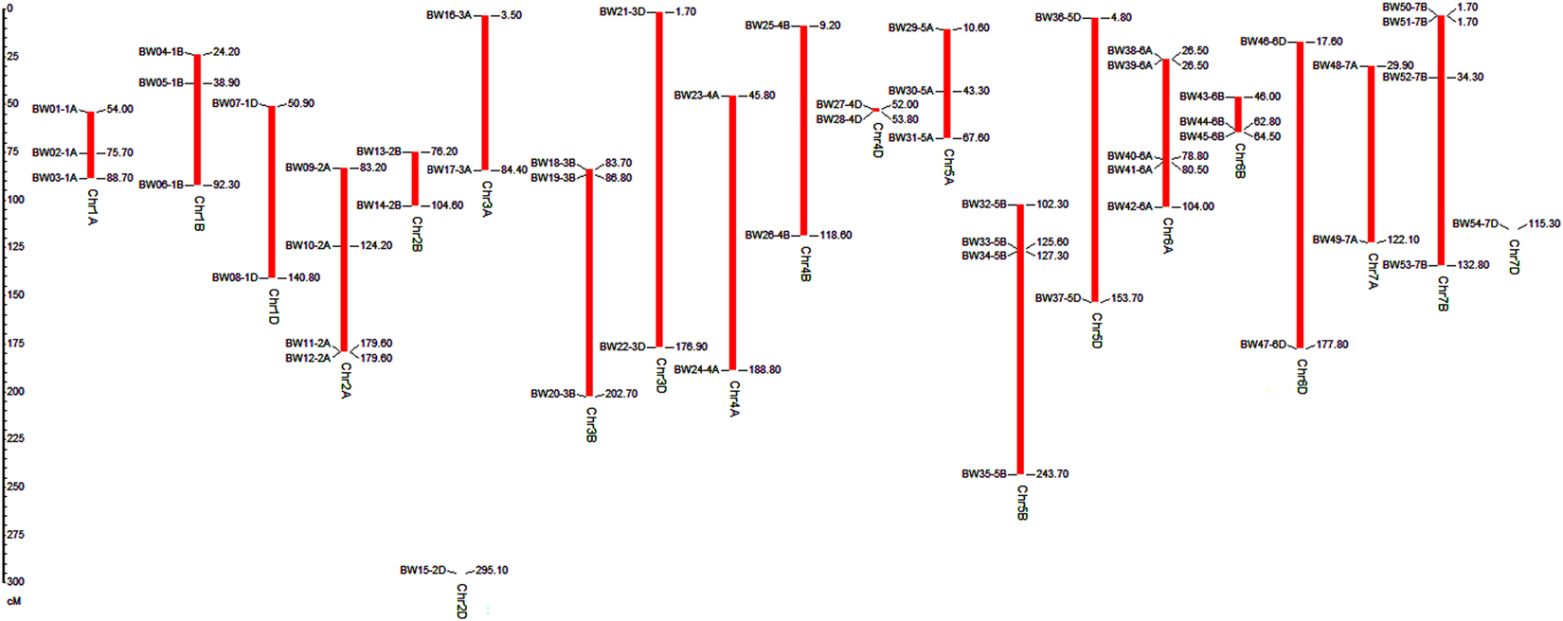
Relative position based on the framework markers position of 54 SNP across the 21 chromosomes. Ruler on the left side denotes centiMorgan (cM) distance and horizontal lines across the chromosomes indicate locus positions on each chromosome.

**Figure 2:**
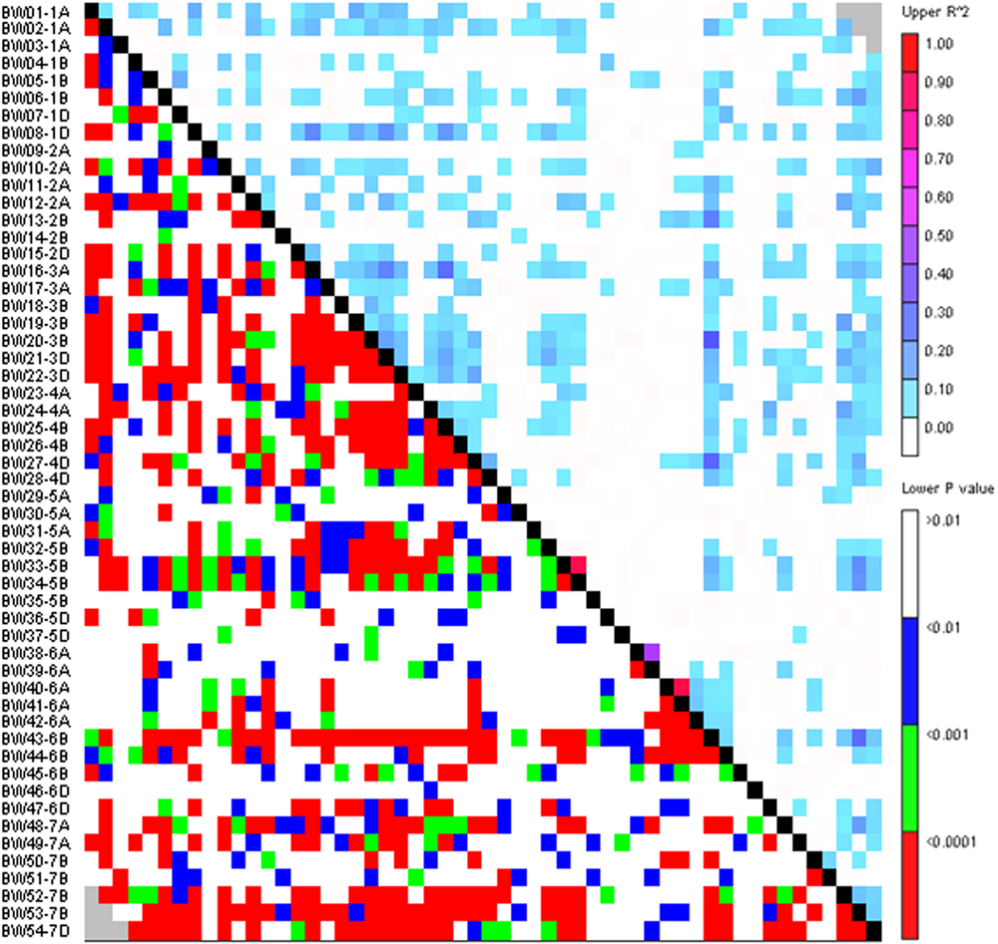
Heat map of linkage disequilibrium (LD) value (R^2^) across the wheat 21 chromosomes measured with 54 SNPs. Markers were ordered on the *x* and *y* axes based on genomic location so that each cell of the heat map represents a single marker pair. The R^2^ values for each marker pair are on the bottom half of the heat map and are represented by shades of colour from 0.0 (white) increasing in darkness in equal increments of 0.1 to 1.0 (red). The p-values of each R^2^ estimate are on the top half of the heat map and are represented by shades of colors from non-significant (p > 0.01; white) highly significant (p < 0.0001; red).

Of the total 16065 SNPs, we identified 54 SNP markers which singled out each of the 368 genotypes used in present study. These markers were distributed throughout the wheat genome (Fig. 1). Each chromosome had two or more markers except chromosome 2D and 7D having one SNP marker. Chromosome 6A had highest five markers. The MAF of the SNP ranged from 0.0976 to 0.5 with average MAF 0.4271. The gene diversity across 54 loci ranged from 0.1761 to 0.5 with average 0.4764. All the SNP markers were having PIC value of >0.3 with average PIC value 0.3615, except two markers on chromosome 5D, namely, AX-95632832 (PIC 0.1606) and AX-94534026 (PIC 0.2506). All the 54 SNP loci were highly informative as they behave independently having loose LD among them (mean *R*^2^ = 0.03) except a pair of markers on chromosome 5B and 6A (Fig. 2). LD can be computed to exclude large number of SNPs (minimization of SNP number). Such approach can select SNPs from each haploblock which are segregating independently. Similar approach has been successfully used in wheat^17,18^ and *Plasmodium*^19^.

Present finding of SNP based differentiation of 368 wheat genotypes confirms the earlier reports that SNP markers have ability to estimate diversity and relatedness in various crops^7^. Genetic resources available in gene banks need detailed characterization so as to enable breeders to utilize them more efficiently. Pure lines or inbreds in crops often contain a multitude of genetically very similar cultivars that require use of highly robust SNP markers for such discrimination. SNP arrays have been efficiently used in several species for discriminating individuals, understanding relatedness across genomes such as *Plasmodium falciparum*^19^, *Mycobacterium tuberculosis*^20^ and crops^17,21–23^. In earlier report, a set of 43 SNPs were unable to differentiate 15.2% of the wheat cultivars because of close relationships among the Chinese accessions^17^. Moreover, these sets of SNP loci are more informative having mean R^2^ = 0.03, in comparison to the earlier report^17^.

Our findings of differentiation of 368 Indian wheat varieties by 54 SNP markers are well in terms of number of varieties and markers in tune of other reports. For example, differentiation of 429 wheat varieties differentiation by 43 SNP^17^, 537 varieties of potato by 50 SNPs^24^, 137 soybean varieties differentiation by 20 SNPs^25^, 121 hop crop varieties differentiation by 7 SNPs^12^, grape variety differentiation by 2–3 SNP/per chromosome^13^, cotton varieties differentiation by 23 core SNP markers^26^ and maize variety differentiation by UPOV using 16 SNP markers^27^. The SNPs identified in our study can be converted into user friendly genotyping assay like KASP/ CASP.

### Phylogenetic tree construction

**Figure 3:**
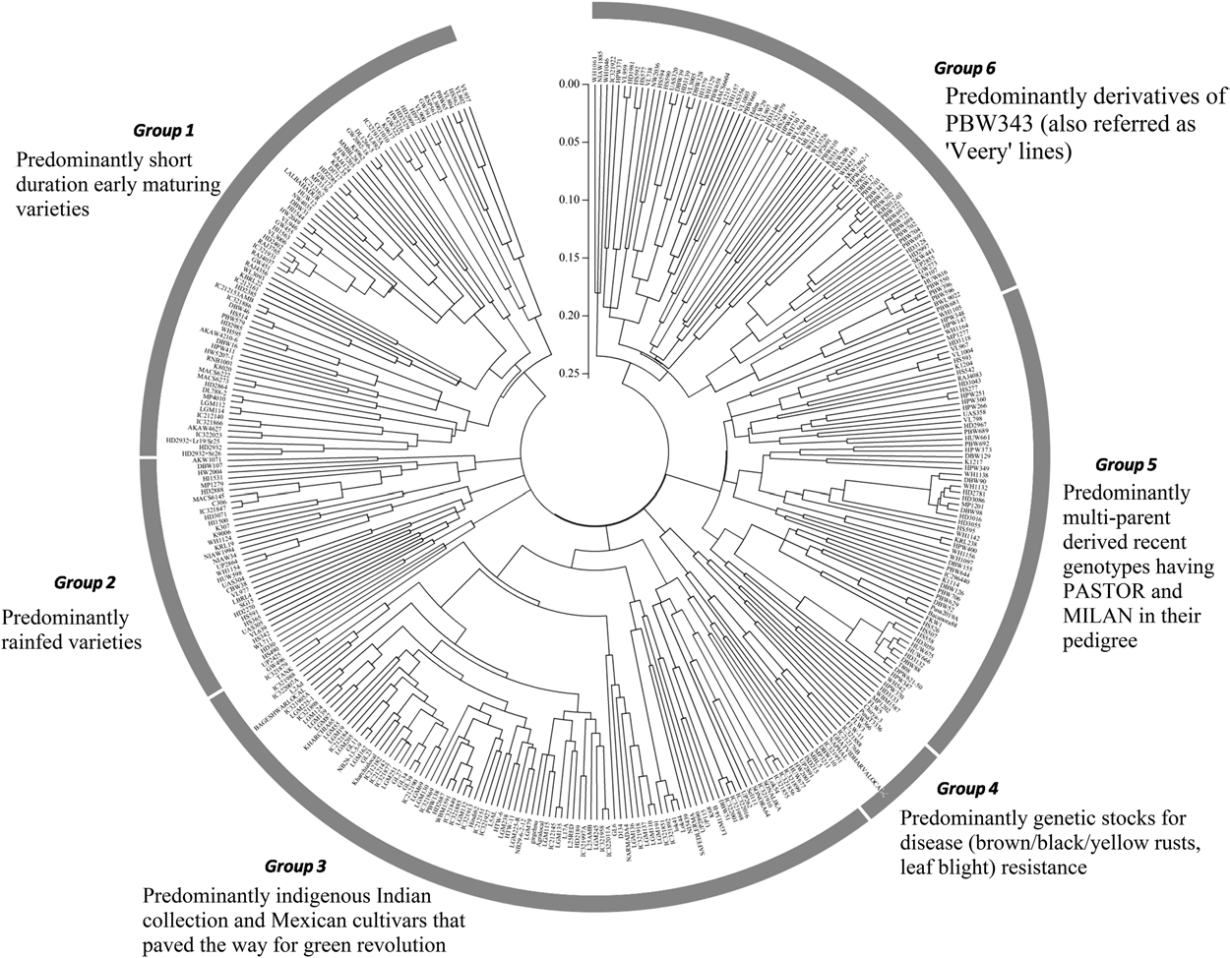
Phylogenetic analysis of 368 wheat genotypes based on the breeder’s 35 K genotyping array. A neighbor-joining (NJ) tree was constructed using identified 54 SNP markers.

Phylogenetic tree constructed using 54 SNPs were found in conformity with their pedigree. There were six clusters representing pre-dominant group of varieties having some common traits or origin or pedigree. They represent predominantly short duration early maturing varieties (cluster 1), predominantly rainfed varieties (cluster 2), predominantly indigenous Indian collection and Mexican cultivars that paved the way for green revolution (cluster 3), predominantly genetic stocks for disease (brown/black/yellow rusts, leaf blight) resistance (cluster 4), predominantly multi-parent derived recent genotypes having PASTOR and MILAN in their pedigree (cluster 5) and predominantly derivatives of PBW343 (‘Veery’ line) (cluster 6) (Fig. 3, Table 1). It further confirms the efficacy and validity of tree constructed, endorsing validity of our approach.

**Table 1:** List of SNP markers used for distinguishing 368 wheat genotypes along with minor allele frequencies (MAF), PIC and genetic diversity

Marker	Assigned code	Chromosome*	Consensus position (cM)*	MAF	Gene Diversity	PIC	Allele
AX-94614591	BW01–1A	1A	54.04	0.4743	0.4987	0.3743	T/G
AX-94548062	BW02–1A	1A	75.74	0.4058	0.4823	0.3660	T/C
AX-94491525	BW03–1A	1A	88.71	0.3983	0.4793	0.3644	A/G
AX-94888336	BW04–1B	1B	24.22	0.4738	0.4986	0.3743	A/G
AX-94755340	BW05–1B	1B	38.86	0.4753	0.4988	0.3744	A/G
AX-94986554	BW06-1B	1B	92.32	0.4932	0.4999	0.3750	A/C
AX-94803245	BW07-1D	1D	50.88	0.3856	0.4738	0.3616	T/C
AX-95070278	BW08-1D	1D	140.84	0.4082	0.4832	0.3664	A/G
AX-94476558	BW09-2A	2A	83.23	0.3945	0.4777	0.3636	A/G
AX-94496990	BW10-2A	2A	124.18	0.3631	0.4625	0.3556	T/C
AX-95217784	BW11-2A	2A	179.61	0.4835	0.4995	0.3747	A/G
AX-94694991	BW12-2A	2A	179.61	0.4033	0.4813	0.3655	T/C
AX-94441179	BW13-2B	2B	76.24	0.4632	0.4973	0.3736	T/C
AX-95628947	BW14-2B	2B	104.59	0.3721	0.4673	0.3581	A/G
AX-94589168	BW15-2D	2D	295.13	0.3179	0.4337	0.3397	T/C
AX-95023272	BW16-3A	3A	3.45	0.4959	0.5000	0.3750	A/G
AX-94664169	BW17-3A	3A	84.43	0.4973	0.5000	0.3750	A/G
AX-94693058	BW18-3B	3B	83.69	0.3817	0.4720	0.3606	A/C
AX-94975644	BW19-3B	3B	86.82	0.4109	0.4841	0.3669	A/G
AX-94704465	BW20-3B	3B	202.68	0.4675	0.4979	0.3739	T/C
AX-94609368	BW21-3D	3D	1.69	0.4914	0.4999	0.3749	A/G
AX-94681475	BW22-3D	3D	176.91	0.3736	0.4681	0.3585	A/C
AX-94795024	BW23-4A	4A	45.84	0.4563	0.4962	0.3731	C/G
AX-95020717	BW24-4A	4A	188.85	0.4538	0.4957	0.3729	T/G
AX-94575968	BW25-4B	4B	9.19	0.4659	0.4977	0.3738	T/C
AX-94522843	BW26-4B	4B	118.61	0.4000	0.4800	0.3648	T/C
AX-94971372	BW27-4D	4D	51.97	0.4823	0.4994	0.3747	T/C
AX-94728173	BW28-4D	4D	53.82	0.3424	0.4503	0.3489	T/G
AX-94438106	BW29-5A	5A	10.64	0.4819	0.4993	0.3747	A/T
AX-94686942	BW30-5A	5A	43.27	0.3590	0.4602	0.3543	A/G
AX-95630073	BW31-5A	5A	67.62	0.4918	0.4999	0.3749	A/G
AX-94816812	BW32-5B	5B	102.31	0.2557	0.3806	0.3082	T/C
AX-94847013	BW33-5B	5B	125.63	0.4850	0.4996	0.3748	A/G
AX-95241690	BW34-5B	5B	127.3	0.4913	0.4998	0.3749	T/C
AX-94727602	BW35-5B	5B	243.74	0.5000	0.5000	0.3750	C/G
AX-94534026	BW36-5D	5D	4.8	0.1789	0.2937	0.2506	T/C
AX-95632832	BW37-5D	5D	153.73	0.0976	0.1761	0.1606	A/C
AX-94507146	BW38-6A	6A	26.51	0.4076	0.4829	0.3663	T/G
AX-95230097	BW39-6A	6A	26.51	0.4849	0.4995	0.3748	A/G
AX-95229606	BW40-6A	6A	78.85	0.4709	0.4983	0.3742	A/C
AX-94437335	BW41-6A	6A	80.46	0.4850	0.4996	0.3748	A/G
AX-94551315	BW42-6A	6A	103.98	0.4206	0.4874	0.3686	T/C
AX-94699925	BW43-6B	6B	45.96	0.3071	0.4256	0.3350	T/C
AX-94986476	BW44-6B	6B	62.83	0.4959	0.5000	0.3750	A/G
AX-95160166	BW45-6B	6B	64.51	0.4860	0.4996	0.3748	T/G
AX-95130119	BW46-6D	6D	17.65	0.4573	0.4964	0.3732	C/G
AX-94388518	BW47-6D	6D	177.76	0.4971	0.5000	0.3750	T/G
AX-94417618	BW48-7A	7A	29.9	0.4973	0.5000	0.3750	A/C
AX-95080011	BW49-7A	7A	122.12	0.4390	0.4926	0.3713	A/T
AX-94848356	BW50-7B	7B	1.72	0.4986	0.5000	0.3750	C/G
AX-95121721	BW51-7B	7B	1.72	0.4802	0.4992	0.3746	T/G
AX-95004702	BW52-7B	7B	34.33	0.4571	0.4963	0.3731	A/G
AX-94431804	BW53-7B	7B	132.83	0.4114	0.4843	0.3670	T/C
AX-94861586	BW54-7D	7D	115.25	0.3945	0.4777	0.3636	C/G
Mean				0.4271	0.4764	0.3615	

^*^Chromosomal position as per consensus map available on CerealsDB (http://www.cerealsdb.uk.net/cerealgenomics/CerealsDB/indexNEW.php)

In the present study, 54 SNP markers were effective enough to differentiate 368 spring genotypes of bread wheat. These markers were able to distinguish the cultivars derived from common lineage such as DPW621-50, DBW88 and HD3059; HD3016, WH1132, WH1138. A summary of genotypes falling in various groups along with the predominant progenitor is mentioned in Table 2.

**Table 2:** Predominant progenitors identified in different groups of wheat genotypes.

SN	Groups of genotypes	Genotypes	Predominant progenitor/comments
1	Predominantly short duration early maturing varieties	K8962, HD2285, Raj3765, DBW 16, MP3336	HD2160
		GW322, MP3336, GW173	GW173
		VL802, VL804, HS562	PBW65
2	Predominantly rainfed varieties	HD2888, HW2004, MACS6145	C 306
		HI1531, HS365, K9006, K307	BLUEBIRD
3	Predominantly indigenous Indian collection and Mexican cultivars that paved the way for green revolution	Kharchia Local, Karchia65, IC212184	Kharchia
		Sonalika, SONORA64, Safed Lerma, UP262, Sel111, HW2001	Sonalika
4	Predominantly genetic stocks for disease (brown/black/yellow rusts, leaf blight) resistance	FLW3, FLW5, FLW11	Multiple rust resistance
5	Predominantly multi-parent derived recent genotypes having PASTOR and MILAN in their pedigree	DPW621-50, DBW88, HD3059	Common pedigree
		HD3016, WH1132, WH1138	Common pedigree
		DBW98, DBW129, HPW400, WH1164, WH1156, HUW661, HPW349	PASTOR
		HUW675, HUW666, HPW373, HD3133, MP1201, HS507, HS542, WH1105	Milan
6	Predominantly derivatives of PBW343 (also referred as ‘Veery’ lines)	PBW343, PBW596, PBW502, VL907, PBW723, PBW693, PBW722, KB2012-03, FLW30, FLW29	PBW343

### Putative role of genes having varietal signature SNP allele

Wheat Breeders’ Array (35 K) designed from 820 K array representing highly informative SNPs have direct implications for wheat breeders especially interested in comparing hexaploid germplasm^28^. SNPs identified in our study having high PIC value distributed on all the 21 wheat chromosomes were also found to possess putative functionality for different biological functions. Out of 54 SNPs, 10 were from non-coding region, thus we obtained putative function of remaining 44 SNPs of the coding regions. Functions of these genes were related to various traits such as flowering, cold acclimatization, water logging, photosynthesis and carbohydrate metabolism, drought/salt/aluminium stress tolerance, seed dormancy and disease resistance. These SNPs were from genes that encodes serine-threonine protein kinases, thiamine pyrophosphokinase 2, alpha-tubulins, methyl-binding domain proteins (MBD), *MOTHER OF FT AND TFL1* (*MFT*), ETERNAL TAPETUM 1 (EAT1), *sn*-glycerol-3-phosphate acyltransferase, phosphoserine aminotransferase, enolase, glucan endo-1,3-beta-glucosidase, receptor protein kinase TMK1, *NAC* transcription factor, aluminum-activated malate transporter 1 (*ALMT1*) and ABC transporters (Supplementary Table 1) proteins which are involved in important biological processes.

### Development of model web-based variety identification system

We report here the first model of crop variety identification system using SNP array using *Triticum aestivum* data in form of VIS*Ta* (http://webtom.cabgrid.res.in/vista/) tool which is based on minimum 54 SNPs and 36 DUS features differentiating >350 varieties. Data/ query can be uploaded and submitted in.txt or excel format or as QR code image in.jpeg format using PC, tablet or mobile device. This tool searches in all the 368 varieties across all the 54 SNPs to find the related varieties with similarity frequencies of each. User can also compare these varieties with 36 DUS features as well as 54 SNPs by checking the box. These tools can be of greater relevance for wheat breeders for *in silico* and rapid identification of varieties based on DUS features and SNPs. The full information of the related varieties can be viewed by putting the cursor on the graphical view.

**Figure 4:**
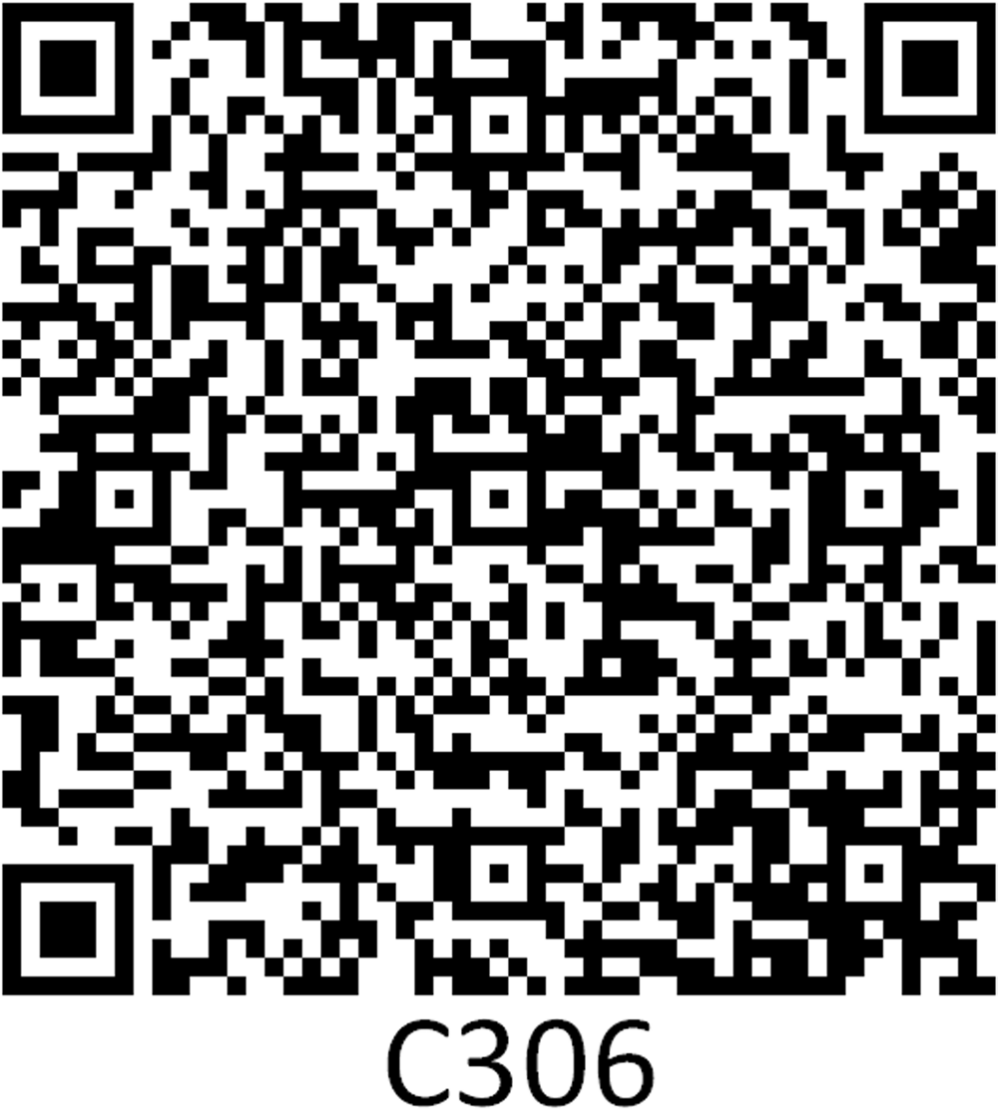
Barcode (2D) of a representative variety of wheat used in the present study.

SNP barcode provides a tool to discriminate very closely related accessions, traceability of minor crops in food supply chain, commercial frauds and dangerous substitutions^29^. Barcode of the selected 368 genotypes have been developed. Figure 4 depicts barcode of representative variety of wheat used in the present study. In some cases such varietal differentiation has been reported to increase the price tag value of wheat in the extent >2-folds in domestic and international market. For instance, Sharbati group of Indian wheat varieties like C306, HI 1500, HI 1531, HI 1544, MP 3211 etc fetch better return to farmers due to higher demand/ premium tag of the variety due to softness and taste of flat bread (*chapati*).

The developed model server, *VISTa* using varietal genomic data of wheat can be implemented in various other crops. While selecting SNP for varietal signature development, few other points must be considered. All SNP sets are not assayable due to genomic complexity and sequence dependent multiplexing parameters^30^. For example, in case of wheat, KASP assay development only 80% of SNP were assayable^31^. SNP present in intergenic or intronic region should be avoided as it might be casually associated, rather than causally. Similarly, silent mutation should be avoided as it is devoid of SAAP (single amino acid polymorphism). Preference should be given to SNPs present in coding region as they exhibit greater stability due to slow mutation rate and more likely to affect changes in the protein associated with phenotypic difference among varieties^32^. DUS feature associated SNP should be preferred^33^. Functional SNP selection offers the advantage due to their potential effect on plant phenotype differences^34^, for example, a non-sense mutation SNP in anthocyanidin synthase gene associated with phenotype uniqueness like yellow pigmentation in raspberry variety has been successfully used^35^. Similar functional SNP associated with DUS feature in barley is also reported^36^.

Our approach can be a model for availability of allelic data in public domain obviating the need of genotyping data generation by multiple users/countries thus would be more logical and economic. In the era of digital communication using hand held communication devices, the present developed mobile app can further popularize this approach of server having huge data of SNP array and varietal signature for remote accessibility and rapid use. Such model user friendly tool can be popular for other crops also as it does not require computational expertise at user end. In era of globalization and best use of germplasm across country, variety identification system can play role in management of germplasm and issues of access benefit sharing (ABS).

## Conclusion

A model web server has been developed successfully for crop variety identification using throughput SNP data of >350 wheat varieties by 35 K SNP chip. VIS*Ta* is world’s first web server for variety identification of any crop using SNP data. In order to make cost effective and rapid genotyping, SNP varietal signature has been successfully made by reduction of SNP up to 54 without compromising identification of >350 varieties. These 54 SNPs based phylogenetic tree confirms six major trait based clusters of Indian wheat varieties along with their pedigree. This tool can also generate QR code to facilitate bar-coding of each variety required for germplasm management. This approach can overcome on limitation of phenotypic data based variety identification by supplementation of molecular data. This server can not only be a model for other crops but can also be used for DUS varietal testing, dispute resolution of sovereignty and ABS issues which is not very uncommon in germplasm movement and improvement in the endevour of wheat productivity and management.

## Supplementary information

Supplementary Table 1

## Data Availability

http://webtom.cabgrid.res.in/vista.
