# Supplementary material for: Development of model web-server for crop variety identification using throughput SNP genotyping data
Source: Sci Rep. 2019 Mar 26;9:5122. doi: 10.1038/s41598-019-41204-2 (PMC6435650; doi:10.1038/s41598-019-41204-2)
Supplement: Supplementary file 1 — Supplementary Table 1 [file 41598_2019_41204_MOESM1_ESM.doc]

**Development of model web-server for crop variety identification using throughput SNP genotyping data**

Rajender Singh^1,#^, MA Iquebal^2,#^, CN Mishra^1^, Sarika Jaiswal^2^, Deepender Kumar^1^, Nishu Raghav^1^, Surinder Paul^1^, Sonia Sheoran^1^, Pradeep Sharma^1^, Arun Gupta^1^, Vinod Tiwari^1^, UB Angadi^2^, Neeraj Kumar^2^, Anil Rai^2^, GP Singh^1^, Dinesh Kumar^2^,* and Ratan Tiwari^1^,*

^1^ICAR-Indian Institute of Wheat & Barley Research, Karnal-132001, Haryana, India

^2^ICAR- Indian Agricultural Statistics Research Institute, New Delhi-110012, India

**Supplementary Table 1**: Putative functions of some of the SNP markers selected for DNA barcoding in wheat

| **S.no.** | **Marker** | **SNP Type** | **Gene** | **Accession No.** | **Putative Function** | **References*** |
| --- | --- | --- | --- | --- | --- | --- |
| 1 | AX-94614591 | K | [Ubiquinone biosynthesis O-methyltransferase](https://blast.ncbi.nlm.nih.gov/Blast.cgi#alnHdr_1149842840) | [XM_020321579.1](https://www.ncbi.nlm.nih.gov/nucleotide/1149842840?report=genbank&log$=nucltop&blast_rank=1&RID=SK060DS8014) | drought responsiveness | 1 |
| 2 | AX-94548062 | Y | [1-acyl-sn-glycerol-3-phosphate acyltransferase 5](https://blast.ncbi.nlm.nih.gov/Blast.cgi#alnHdr_1149844853) | [XM_020322338.1](https://www.ncbi.nlm.nih.gov/nucleotide/1149844853?report=genbank&log$=nucltop&blast_rank=1&RID=SK0FPWN7014) | photosynthetic rate under high temperature in wheat | 2 |
| 3 | AX-94491525 | R | Efflux RND (resistance nodulation cell division) transporter permease subunit | - | mediated transport of substrates out of cell | 3 |
| 4 | AX-94755340 | R | [Alpha tubulin-2B (TUBA-2B)](https://blast.ncbi.nlm.nih.gov/Blast.cgi#alnHdr_90289599) | [DQ435661.1](https://www.ncbi.nlm.nih.gov/nucleotide/90289599?report=genbank&log$=nucltop&blast_rank=1&RID=SK2TCWX7015) | Cold acclimation | 4 |
| 5 | AX-94986554 | M | glucan 1,4-alpha-glucosidase | - | dough behavior (flour & bread making quality) of wheat | 5 |
| 6 | AX-94803245 | Y | [Serine/threonine-protein kinase WNK2](https://blast.ncbi.nlm.nih.gov/Blast.cgi#alnHdr_1149790074) | [XM_020301110.1](https://www.ncbi.nlm.nih.gov/nucleotide/1149790074?report=genbank&log$=nucltop&blast_rank=1&RID=SK37Z5J8014) | Flowering | 6 |
| 7 | AX-94496990 | Y | [Transcription factor EAT1-like](https://blast.ncbi.nlm.nih.gov/Blast.cgi#alnHdr_1149699270) | [XM_020296560.1](https://www.ncbi.nlm.nih.gov/nucleotide/1149699270?report=genbank&log$=nucltop&blast_rank=1&RID=SNBMUH0P014) | Male sterility | 7 |
| 8 | AX-94694991 | Y | NRT1 PTR FAMILY -like | SPT21095 | nitrate availability and grain filling in wheat | 8 |
| 9 | AX-94441179 | Y | [Thiamine pyrophosphokinase 2](https://blast.ncbi.nlm.nih.gov/Blast.cgi#alnHdr_1149753100) | [XM_020343194.1](https://www.ncbi.nlm.nih.gov/nucleotide/1149753100?report=genbank&log$=nucltop&blast_rank=1&RID=SNC1F3ZN014) | Photosynthesis and carbohydrate metabolism | 9 |
| 10 | AX-95628947 | R | HSP1 gene | EMS53542.1 | Associated with wheat fungal leaf blotch | 10 |
| 11 | AX-94589168 | Y | beta-fructofuranosidase | AVY91565.1 | Associated with carbohydrate metabolism in stripe rust of wheat | 11 |
| 12 | AX-95023272 | R | flowering time control FCA-like isoform X1 | OIT35882 | Flowering time control in wheat | 12 |
| 13 | AX-94664169 | R | hypothetical protein TRIUR3_13242 | EMS48155 | Zn finger protein associated in wheat with abiotic stress | 13 |
| 14 | AX-94693058 | M | [ABC transporter B family member 4-like](https://blast.ncbi.nlm.nih.gov/Blast.cgi#alnHdr_1149822675) | [XM_020313950.1](https://www.ncbi.nlm.nih.gov/nucleotide/1149822675?report=genbank&log$=nucltop&blast_rank=2&RID=SNB4V1Z7014) | Auxin mediated lateral root and root hair development | 14 |
| 15 | AX-94975644 | R | ABC transporter F family member 4-like | [XM_020307314.1](https://www.ncbi.nlm.nih.gov/nucleotide/1149806230?report=genbank&log$=nucltop&blast_rank=1&RID=SNAZD02G014) | grain formation and mycotoxin tolerance | 15 |
| 16 | AX-94704465 | Y | [Glucan endo-1,3-beta-glucosidase GV](https://blast.ncbi.nlm.nih.gov/Blast.cgi#alnHdr_1149705966) | [XM_020323513.1](https://www.ncbi.nlm.nih.gov/nucleotide/1149705966?report=genbank&log$=nucltop&blast_rank=2&RID=SNAJYYXV014) | Drought tolerance | 16 |
| 17 | AX-94609368 | R | [MFT-3D](https://blast.ncbi.nlm.nih.gov/Blast.cgi#alnHdr_913375214) | [AB924663.1](https://www.ncbi.nlm.nih.gov/nucleotide/913375214?report=genbank&log$=nucltop&blast_rank=1&RID=SNA46EB2015) | Seed dormancy/ pre-harvest sprouting | 17 |
| 18 | AX-94681475 | M | aceous RNase P 2-like | BAS76401 | cleavage of some mRNAs and non-coding RNAs in wheat | 18 |
| 19 | AX-94795024 | S | [Phosphoserine aminotransferase 1](https://blast.ncbi.nlm.nih.gov/Blast.cgi#alnHdr_1149738402) | [XM_020337679.1](https://www.ncbi.nlm.nih.gov/nucleotide/1149738402?report=genbank&log$=nucltop&blast_rank=1&RID=SNMRXARV014) | Serine biosynthetic pathway in plastids | 19 |
| 20 | AX-95020717 | K | ribosomal RNA large subunit methyltransferase N | CDE86982 | wheat seedling development | 20 |
| 21 | AX-94575968 | Y | O-methyltransferase ZRP4 | NP_001140810 | lodging resistance(stem/culm mechanical strength) and Sharp eyespot fungal disease resistance of wheat | 21 |
| 22 | AX-94522843 | Y | family transcriptional regulator | RLE69119 | flocculation and wheat root surface colonization | 22 |
| 23 | AX-94971372 | Y | [Aluminum-activated malate transporter 1](https://blast.ncbi.nlm.nih.gov/Blast.cgi#alnHdr_1149809469) | [XM_020308558.1](https://www.ncbi.nlm.nih.gov/nucleotide/1149809469?report=genbank&log$=nucltop&blast_rank=1&RID=TMU238UV014) | Aluminum tolerance | 23 |
| 24 | AX-94728173 | K | [Receptor protein kinase TMK1-like](https://blast.ncbi.nlm.nih.gov/Blast.cgi#alnHdr_1149805675) | [XM_020307089.1](https://www.ncbi.nlm.nih.gov/nucleotide/1149805675?report=genbank&log$=nucltop&blast_rank=1&RID=TMUZCKCG016) | Auxin signal transduction in root and potentially shoot development;  biotic and abiotic (heat, drought, and salt) stresses in wheat | 24, 25 |
| 25 | AX-94438106 | W | serine threonine receptor-like cytoplasmic kinase | XM_020345073.1 | Biotic stress resistance in wheat | 26 |
| 26 | AX-94686942 | R | [TaENO-a enolase](https://blast.ncbi.nlm.nih.gov/Blast.cgi#alnHdr_461744055) | [KC342469.1](https://www.ncbi.nlm.nih.gov/nucleotide/461744055?report=genbank&log$=nucltop&blast_rank=1&RID=TN8J8S69016) | Low temperature tolerance | 27 |
| 27 | AX-95630073 | R | Heavy metal-associated isoprenylated plant protein 35-like | [XM_020340390.1](https://www.ncbi.nlm.nih.gov/nucleotide/1149745806?report=genbank&log$=nucltop&blast_rank=1&RID=TN8TUDTZ016) | drought and metal stress tolerance in wheat | 28 |
| 28 | AX-94816812 | Y | [MBD2 mRNA](https://blast.ncbi.nlm.nih.gov/Blast.cgi#alnHdr_134285525) | [EF469225.1](https://www.ncbi.nlm.nih.gov/nucleotide/134285525?report=genbank&log$=nucltop&blast_rank=3&RID=TN90FA34016) | Salt stress tolerance | 29 |
| 29 | AX-94847013 | G | F Box-protein gene | XP_020194275.1 | Wheat heat tolerance | 30 |
| 30 | AX-95241690 | Y | Trypsin-like peptidase(TLP) | - | Wheat leaf apoplasts. | 31 |
| 31 | AX-94534026 | Y | [Serine/arginine repetitive matrix protein 1-like](https://blast.ncbi.nlm.nih.gov/Blast.cgi#alnHdr_1149837804) | [XM_020319586.1](https://www.ncbi.nlm.nih.gov/nucleotide/1149837804?report=genbank&log$=nucltop&blast_rank=1&RID=TN9YAEY2014) | Abnormal spike development | 32 |
| 32 | AX-95632832 | M | [Syntaxin-132-like](https://blast.ncbi.nlm.nih.gov/Blast.cgi#alnHdr_1149706989) | [XM_020324019.1](https://www.ncbi.nlm.nih.gov/nucleotide/1149706989?report=genbank&log$=nucltop&blast_rank=1&RID=TNA6N0RP014) | Disease resistance | 33 |
| 33 | AX-95230097 | R | RpsA (Ribosomal protein S1) | XM_020343821.1 | Associated with laminin receptor involved in growth and differentiation in wheat | 34 |
| 34 | AX-94437335 | R | [Acyltransferase-like protein](https://blast.ncbi.nlm.nih.gov/Blast.cgi#alnHdr_1149710632) | [XM_020325799.1](https://www.ncbi.nlm.nih.gov/nucleotide/1149710632?report=genbank&log$=nucltop&blast_rank=1&RID=TRDCJ3B6014) | triacylglycerol (TAG) synthesis in developing wheat embryos | 35 |
| 35 | AX-94699925 | Y | transcription termination factor mitochondrial-like | EMS59998 | Post transcriptoional regulation/RNA editing in wheat/ | 36 |
| 36 | AX-95160166 | K | alpha/beta hydrolase | WP_051732828 | Abiotic Stress tolerance in wheat | 37 |
| 37 | AX-95130119 | S | [NAC domain-containing protein 78-like](https://blast.ncbi.nlm.nih.gov/Blast.cgi#alnHdr_1149745611) | [XM_020340304.1](https://www.ncbi.nlm.nih.gov/nucleotide/1149745611?report=genbank&log$=nucltop&blast_rank=1&RID=TRNPA9WE014) | High light stress | 38 |
| 38 | AX-94388518 | K | F Boxprotein gene | XM_020290580 | Wheat heat tolerance | 30 |
| 39 | AX-94417618 | M | [Palmitoyl-acyl carrier protein thioesterase](https://blast.ncbi.nlm.nih.gov/Blast.cgi#alnHdr_1149805539) | [XM_020307020.1](https://www.ncbi.nlm.nih.gov/nucleotide/1149805539?report=genbank&log$=nucltop&blast_rank=2&RID=TRNXS09W014) | drought stress in wheat | 39 |
| 40 | AX-94848356 | S | alanine racemase | WP_066745268 | Wheat seedling growth | 40 |
| 41 | AX-95004702 | R | [Protein OS-9 homolog](https://blast.ncbi.nlm.nih.gov/Blast.cgi#alnHdr_1149759621) | [XM_020345633.1](https://www.ncbi.nlm.nih.gov/nucleotide/1149759621?report=genbank&log$=nucltop&blast_rank=1&RID=TRPW70Y2014) | Salt stress tolerance | **41** |
| 42 | AX-94431804 | Y | [HKT transporter](https://blast.ncbi.nlm.nih.gov/Blast.cgi#alnHdr_982744397) | [KR422355.1](https://www.ncbi.nlm.nih.gov/nucleotide/982744397?report=genbank&log$=nucltop&blast_rank=4&RID=TRPZMW8U016) | Salt stress tolerance;  Na+ transporters and their mechanisms mediating salinity resistance | 42, 43 |
| 43 | AX-94861586 | S | [Glycosyltransferase family 92](https://blast.ncbi.nlm.nih.gov/Blast.cgi#alnHdr_1149704048) | [XM_020319767.1](https://www.ncbi.nlm.nih.gov/nucleotide/1149704048?report=genbank&log$=nucltop&blast_rank=1&RID=TRR5PG2A016) | FHB resistance in wheat | 44 |
| 44 | AX-95217784 | R | Na+/Ca2+ Exchanger Protein (NCX) Gene | XM_020344001.1 | Associated with salt stress in wheat | 45 |

*References of Supplementary Table 1:

1. Wang, X., Cai, X., Xu, C., Wang, Q., & Dai, S. Drought-responsive mechanisms in plant leaves revealed by proteomics. *International journal of molecular sciences*. **17(10),** 1706 (2016).
2. Djanaguiraman, M., Boyle, D. L., Welti, R., Jagadish, S. V. K., & Prasad, P. V. V. Decreased photosynthetic rate under high temperature in wheat is due to lipid desaturation, oxidation, acylation, and damage of organelles. *BMC plant biology*. **18(1),** 55 (2018).
3. Pak, J. E. *et al.,* Structures of intermediate transport states of ZneA, a Zn (II)/proton antiporter. *Proceedings of the National Academy of Sciences.* 201318705 (2013).
4. Ridha Farajalla, M., & Gulick, P. J. The α-tubulin gene family in wheat (Triticum aestivum L.) and differential gene expression during cold acclimation. *Genome*. **50(5),** 502-510 (2007).
5. Barrera, G. N., León, A. E., & Ribotta, P. D. Use of enzymes to minimize the rheological dough problems caused by high levels of damaged starch in starch–gluten systems. *Journal of the Science of Food and Agriculture*. **96(7),** 2539-2546 (2016).
6. Wang Y. *et al.,* The plant WNK gene family and regulation of flowering time in Arabidopsis. Plant Biology. **10,** 548–562 (2008).
7. Niu N., Liang W., Yang X., Jin W., Wilson Z.A., Hu J., Zhang D. EAT1 promotes tapetal cell death by regulating aspartic proteases during male reproductive development in rice. *Nat. Commun*. **4,** 1445 (2013).
8. Buchner, P. & Hawkesford, M. J. Complex phylogeny and gene expression patterns of members of the NITRATE TRANSPORTER 1/PEPTIDE TRANSPORTER family (NPF) in wheat. *Journal of experimental botany*. **65(19),** 5697-5710 (2014).
9. Ajjawi, I., Milla, M. A. R., Cushman, J., & Shintani, D. K. Thiamin pyrophosphokinase is required for thiamin cofactor activation in Arabidopsis. *Plant molecular biology*. ***65*(1-2),** 151-162 (2007).
10. Keon J, Bailey A, Hargreaves J. A group of expressed cDNA sequences from the wheat fungal leaf blotch pathogen, Mycosphaerella graminicola (Septoria tritici). *Fungal Genet Biol.* **29(2)**, 118-33 (2000).
11. Coram TE, Wang M and Chen X. Transcriptome analysis of the wheat–Puccinia striiformis f. sp. tritici interaction. *Molecular Plant Pathology,* **9(2),** 157–169 (2008).
12. Cockram, J. *et al.,* Control of flowering time in temperate cereals: genes, domestication, and sustainable productivity. *Journal of Experimental Botany*. **58(6),** 1231-1244 (2007).
13. Cheuk, A. & Houde, M. Genome wide identification of C1-2i zinc finger proteins and their response to abiotic stress in hexaploid wheat. *Molecular genetics and genomics*. **291(2),** 873-890 (2016).
14. Santelia, D. *et al.,* MDR‐like ABC transporter AtPGP4 is involved in auxin‐mediated lateral root and root hair development. *FEBS letters*. **579(24),** 5399-5406 (2005).
15. Walter, S. *et al.,* A wheat ABC transporter contributes to both grain formation and mycotoxin tolerance. *Journal of experimental botany*. **66(9),** 2583-2593 (2015).
16. Faghani, E. *et al.,* Comparative physiology and proteomic analysis of two wheat genotypes contrasting in drought tolerance. *Journal of proteomics*. **114,** 1-15 (2015).
17. Nakamura, S. *et al.,* A wheat homolog of MOTHER OF FT AND TFL1 acts in the regulation of germination. *The Plant Cell*. tpc-111 (2011).
18. Krehan, M., Heubeck, C., Menzel, N., Seibel, P., & Schön, A. RNase MRP RNA and RNase P activity in plants are associated with a Pop1p containing complex. *Nucleic acids research*. **40(16),** 7956-7966 (2012).
19. Ros, R. *et al.,* Serine biosynthesis by photorespiratory and non‐photorespiratory pathways: an interesting interplay with unknown regulatory networks. *Plant Biology*. **15(4),** 707-712 (2013).
20. Yin, N. *et al.,* Analysis of differential proteins induced by forchlorfenuron in wheat. *Plant molecular biology reporter*. **30(4),** 949-956 (2012).
21. Wang, M. *et al.,* A wheat caffeic acid 3-O-methyltransferase TaCOMT-3D positively contributes to both resistance to sharp eyespot disease and stem mechanical strength. *Scientific reports*. **8**, 6543 (2018).
22. Pereg-Gerk, L., Paquelin, A., Gounon, P., Kennedy, I. R., & Elmerich, C. A transcriptional regulator of the LuxR-UhpA family, FlcA, controls flocculation and wheat root surface colonization by Azospirillum brasilense Sp7. *Molecular plant-microbe interactions*. **11(3),** 177-187 (1998).
23. Sasaki, T. *et al.,* A wheat gene encoding an aluminum‐activated malate transporter. *The Plant Journal*. **37(5),** 645-653 (2004).
24. Dai, N., Wang, W., Patterson, S. E., & Bleecker, A. B. The TMK subfamily of receptor-like kinases in Arabidopsis display an essential role in growth and a reduced sensitivity to auxin. *PLoS One*. **8(4),** e60990 (2013).
25. Sharma, S., Kumar, R., Mendu, V., Singh, K., & Upadhyay, S. K. Genomic dissection and expression profiling revealed functional divergence in Triticum aestivum leucine rich repeat receptor like kinases (TaLRRKs). *Frontiers in plant science*. **7,** 1374 (2016).
26. Yang, K. *et al.,* Isolation and characterization of a novel wheat cysteine-rich receptor-like kinase gene induced by Rhizoctonia cerealis. *Scientific reports*. **3,** 3021 (2013).
27. Sharma, P., Ganeshan, S., Fowler, D. B., & Chibbar, R. N. Characterisation of two wheat enolase cDNA showing distinct patterns of expression in leaf and crown tissues of plants exposed to low temperature. *Annals of applied biology*. **162(2),** 271-283 (2013).
28. Singh, S. P. *et al.,* Comparative transcriptional profiling of two wheat genotypes, with contrasting levels of minerals in grains, shows expression differences during grain filling. *PloS one*. **9(11),** e111718 (2014).
29. Hu, Z. *et al.,* Expression divergence of TaMBD2 homoeologous genes encoding methyl CpG-binding domain proteins in wheat (Triticum aestivum L.). *Gene*. **471(1),** 13-18 (2011).
30. Li Q, Wang W, Wang W, *et al.,* Wheat F-Box Protein Gene TaFBA1 Is Involved in Plant Tolerance to Heat Stress. *Front Plant Sci*. **9**, 521 (2018).
31. Segarra CI, Casalongué CA, Pinedo ML, Ronchi VP, Conde RD. A germin-like protein of wheat leaf apoplast inhibits serine proteases. J Exp Bot. 2003 May;54(386):1335-41.
32. Zhu, X. X. *et al.,* Transcriptome analysis for abnormal spike development of the wheat mutant dms. *PloS one*. **11(3),** e0149287 (2016).
33. Kalde, M., Nühse, T. S., Findlay, K., & Peck, S. C. The syntaxin SYP132 contributes to plant resistance against bacteria and secretion of pathogenesis-related protein 1. *Proceedings of the National Academy of Sciences*. **104(28),** 11850-11855 (2007).
34. DiGiacomo V, and Meruelo D. Looking into laminin receptor: critical discussion regarding the non-integrin 37/67-kDa laminin receptor/RPSA protein. *Biol Rev Camb Philos Soc*. **91(2)**, 288–310 (2016).
35. Rodríguez-Sotres, R., Pacheco-Moisés, F., & Black, M. Role of Acyltransferases in the Effect of ABA and Osmoticum on Oil Deposition during Seed Development in *Basic and Applied Aspects of Seed Biology. Current Plant Science and Biotechnology in Agriculture* (eds. Ellis R.H., Black M., Murdoch A.J., Hong T.D.) **30,** 153-158 (Springer, Dordrecht, 1997).
36. Hammani K and Giege P. RNA metabolism in plant mitochondria, *Trends in Plant Science*, **19(6),** 380-390 (2014).
37. Khatri N and Mudgil Y. Hypothesis: NDL proteins function in stress responses by regulating microtubule organization. *Front Plant Sci*. **6**, 947 (2015).
38. Morishita, T. *et al.,* Arabidopsis NAC transcription factor, ANAC078, regulates flavonoid biosynthesis under high-light. *Plant and Cell Physiology.* **50(12),** 2210-2222 (2009).
39. Navabpour, S., Ramazanpour, S.S., Soltanloo, H., & Vakili Bastam, S. Drought stress changed expression profile of some genes in tillering and pollination stages of adult wheat. *Applied Science Reports*. **9(2),** 100-109 (2015).
40. Gamburg K Z, Gluzdo OV, Rekoslavskaya NI.The content of N-malonyl-D-tryptophan in wheat seedlings. *Plant Science*, **88 (1),** 121-124 (1993).
41. Hüttner, S., Veit, C., Schoberer, J., Grass, J., & Strasser, R. Unraveling the function of Arabidopsis thaliana OS9 in the endoplasmic reticulum-associated degradation of glycoproteins. *Plant molecular biology*. **79(1-2),** 21-33 (2012).
42. Ariyarathna, H. C. K., Oldach, K. H., & Francki, M. G. A comparative gene analysis with rice identified orthologous group II HKT genes and their association with Na+ concentration in bread wheat. *BMC plant biology*. **16(1),** 21 (2016).
43. Horie, T., Hauser, F., & Schroeder, J. I. HKT transporter-mediated salinity resistance mechanisms in Arabidopsis and monocot crop plants. *Trends in plant science*. **14(12),** 660-668 (2009).
44. He, Y. *et al*. Genome-wide analysis of family-1 UDP glycosyltransferases (UGT) and identification of UGT genes for FHB resistance in wheat (Triticum aestivum L.). *BMC plant biology*. **18(1),** 67 (2018).
45. Wang P, Li Z, Wei J, Zhao Z, Sun D, and Cui S. A Na+/Ca2+ Exchanger-like Protein (AtNCL) Involved in Salt Stress in Arabidopsis. *J Biol Chem.* **287(53),** 44062–44070 (2012).
